# In-vivo Comparison of ^18^F-FLT uptake, CT Number, Tumor Volume in Evaluation of Repopulation during Radiotherapy for Lung cancer

**DOI:** 10.1038/srep46000

**Published:** 2017-04-07

**Authors:** Xiaoli Zhang, Jinming Yu, Chengming Li, Xindong Sun, Xue Meng

**Affiliations:** 1 Department of Oncology, Renmin Hospital of Wuhan University, Wuhan, China; 2 Department of Radiation Oncology, Shandong Cancer Hospital Affiliated to Shandong University, Shandong Academy of Medical Science, Jinan, China; 3 Shandong Academy of Medical Sciences, Jinan University, Jinan, China

## Abstract

Accelerated repopulation has been observed in various tumors. This study was aimed to evaluate the potential of 3′-deoxy-3′-^18^F-fluorothymidine (^18^F-FLT) uptake and Computed Tomography Number (CTN) in monitoring tumor responses to radiotherapy compared with tumor volume (TV) changes. Tumor bearing nude mice were assigned to either irradiated daily or every second day group and then randomized to 6 sub-groups to receive 0Gy, 6Gy, 12Gy, 18Gy, 24Gy, 36Gy irradiation, respectively. TV was measured every 3 days. ^18^F-FLT micro-PET/CT scans were performed after irradiation being completed. Tumor sections were stained to calculate the immunohistochemical (Ki-67) labeling index (LI). Comparison analysis between FLT uptake parameters, CTNs, VTs and Ki-67 LI results were conducted to determine the correlation. Ki-67 LI increased significantly after 6 times of irradiation at irradiated daily group and after 3 times at irradiated every second day group, suggesting accelerated repopulation. No shrinkage of TV was noticed at two groups during irradiation delivery. Both ^18^F-FLT uptake and CTN increased significantly after irradiation of 12Gy/6f/6d and 6Gy/3f/6d. Comparison analysis found a significant relationship between Ki-67 LI and ^18^F-FLT uptake parameters as well as CTN. Both ^18^F-FLT PET and CT have the potential to reflect the tumor proliferative response during radiation delivery.

Anticancer treatment has translated from simple nonspecific cytotoxic therapy to ever-more-sophisticated specific individualized therapeutics. In the battle with cancer, the imbalance between endless emergence of novel technologies and relatively poor survival become more and more severe. Cancer intrinsic radio-biological or physiological characteristics at the molecular level (metabolism, proliferation, hypoxia, and so on)^1^,^2^,^3^, which were constitutional or acquired during treatment, might have contributed to this phenomenon. Results of studies in both preclinical and clinical settings have revealed that re-proliferation of tumors during the course of irradiation plays an important role in radio-resistance^4^,^5^,^6^. So giving a relatively high irradiation dose to the re-proliferative area at the given time might bring a higher local control rate.

But one of the open issues for adaptive radiation (RT) is to observe the growth of tumor and determine the specific timing of tumor cell re-proliferation in order to trigger adaptive re-plan during the course of fractionated RT delivery. Evaluating the solid tumor volume (TV) during irradiation is the easiest way to detect tumor growth^7^. But the changes of TV often appeared later and not discriminated significantly during fractionated irradiation or other treatment progress^8^. Appearance of various image-guided technologies allows for more noninvasive, precise, quantitative, *in vivo* methods to detect the inter-fraction variations of tumors during RT delivery, ranging from anatomic, histologic changes to metabolic, molecular, functional changes. Among those emerging methods, 3′-deoxy-3′-^18^F-fluorothymidine (^18^F-FLT) position-emission tomography (PET) with the capacity to detect molecular biological characteristics of *in vivo* tumors shows the potential to monitor tumor growth and then address the limitations of conventional response-assessments methods. Ki-67 expression which is strictly correlated with cell proliferation and with the active phases of the cell cycle^9^, has exhibited excellent consistence with FLT uptake in both preclinical^10^,^11^ and clinical researches^12^,^13^. Studies recently conducted in our department also verified the spatial coincidence of ^18^F-FLT PET image and Ki-67 image for human lung adenocarcinoma before and during irradiation. But the relatively low cost-effectiveness of this new technology limits its clinical value, especially in developing countries.

With the analytical capabilities of some traditional imaging modalities (eg. computed tomography [CT], magnetic resonance imaging) being enhanced, quantitative image signatures known as radiomics or radiogenomics come into being and show the potential to extract some novel phenotypic characteristics of tumors from currently available data^14^. CT which has been commonly used to detect and help delineate tumors, is now found to be able to reflect the tumor response especially growth status during treatment. Feng *et al*. have ever investigated CT number (CTN) changes of gross tumor volume (GTV) according to daily diagnostic-quality CT-on-rails acquired during CT-guided intensity modulated radiation therapy in head and neck cancer patients, and found a fair correlation between CTN reduction and radiation doses^15^. This implied the potential of CTN as an early surrogate biomarker to evaluate tumor response. Report from a pilot study conducted by Zhao *et al*. at Memorial Sloan-Kettering Cancer Center revealed that though not much data existed to support the hypothesis that tumor density changes may precede size changes with therapy, ghosted and cystic changes have been observed clinically before the tumor shrinkage^16^.

In this work we conducted a study to investigate the TVs, ^18^F-FLT uptake parameters, CTN changes of tumors according to repetitive PET/CT scans and pathological evaluations for human A549 lung adenocarcinoma tumor-bearing BALB/c nude mice. An effort will be made to examine the relationships between TV regressions, ^18^F-FLT parameters, CTN changes, and delivered radiation doses and to determine whether these changes could potentially help predict the re-proliferation of tumor.

## Material and Methods

### Experimental Design

One hundred and twenty tumor-bearing nude mice were randomized into 2 groups (60 for each group); group 1 was irradiated daily and group 2 was irradiated every second day. The 60 nude mice in each group were randomized to receive 0Gy/0fraction (f), 6Gy/3f, 12Gy/6f, 18Gy/9f, 24Gy/12f, 36Gy/18f, respectively. So there were 10 mice in each sub-group. When the tumors grew to 0.8–1.0 cm, they were locally exposed to fractioned radiotherapy. ^18^F-FLT micro-PET/CT scans were performed immediately after the given dose being received by each group, with ^18^F-FLT parameters and Computed Tomography Number (CTN) being measured. The tumor size was measured every 3 days for all the mice and growth curves were prepared. When image acquisition finished, tumors were excised for immunohistochemical staining. The animal facility and experiments were approved according to Chinese animal welfare regulations. All experimental protocols were approved by the Ethics Committee of Shandong Cancer Hospital Affiliated to Shandong University. All methods were performed in accordance with the relevant guidelines and regulations.

### Animal Model

Female Balb/c nude mice inoculated with A549 tumors were used for the assessments. A549, a human adenocarcinoma cell carcinoma cell line, which was free of mycoplasmas and obtained from Chinese Academy of Sciences Shanghai Institute of Cell Bank, was inoculated subcutaneously into the right hind limbs of 8-wk-old nude mice as single-cell suspensions at a cell density of 2 × 10^6^ in 0.1 ml of phosphate buffer saline (PBS).

### Tumor volume

The tumor volume (TV) was determined by vernier caliper measurements using the following formula: TV = π/6 × l × w^2^ (where “l” represents the longest and “w” the perpendicular tumor axes)^11^. In order to avoid individual variables, all of the tumors were evaluated by the same investigator.

### Radiation Treatment

Irradiation was performed with 6 MeV of x-rays by a linear accelerator (X-RAD 225; Varian Medical Systems, Inc.). The mice were immobilized using plastic tubes fixed to a plastic foamboard with the tumor-bearing leg positioned in the radiation field by a foot-holder distal to the tumor and the rest parts of the mice were protected by a lead. The tumor bearing nude mice were irradiated at room temperature without anesthesia, and local irradiation of the tumors was executed at a fractioned dose of 2 Gy, a commonly used daily fractionated dose in clinical therapy. Different numbers of fractions were delivered to nude mice either once daily or once every second day Monday through Sunday.

### Micro-PET imaging

Inveon DPET (Siemens Preclinical Solutions, Knoxville, TN, USA) was used to perform the Micro-PET scans of tumors. According to the method developed by Grierson^17^, the synthesis of ^18^F-FLT was performed with purities of greater than 99% as previous reported by Yue *et al*.^11^ from the Jiangsu Atomic Energy Laboratory, Jiangsu, China. Every nude was injected with ^18^F-FLT, a median activity of 4.37 MBp (range, 0.68–5.01 MBq) via the tail vein and performed PET scan 60 min after injection of ^18^F-FLT. Anesthesia was maintained with 1.5% isoflurane in 100% oxygen with a flow of 1.5 L/min. Mice were positioned prone on the bed of the micro-PET, with four limbs fixed with tape. A 10-min static single-frame scan was acquired on the small-animal PET camera, and images were reconstructed by OSEM-3D IAW (Siemens Preclinical Solutions, Knoxville, TN, USA). The images were analyzed independently by two experienced nuclear medicine physicians. Quantitative analysis was performed using the standardized uptake value (SUV). The region of interest (ROI) encompassed the entire tumor on all of the transverse PET planes containing the lesion. The reference tissue was defined as a circular region of 5 × 5 mm manually contoured in the lung on the transverse PET image. The ROI was used to calculate the maximum SUV (SUVmax) and the mean SUV (SUVmean) by taking the ratio of maximal or mean tracer uptake in the tumor to the injected activity normalized to body weight. The tumor-to-normal tissue (T/NT) ratio was calculated as tumor SUVmax divided by lung SUVmax^11^.

### Micro-CT imaging

After micro-PET scanning was finished, every nude immediately experienced micro-CT scan through a MicroCAT^®^ II system (Siemens Medical Solutions) with a slice thickness of 0.08 mm, no intervals, and exactly the same localization as PET scanning. For the sake of consistency, reproducible scanning conditions (with an x-ray tube voltage peak of 90 kV, 200 μA; spatial resolution of 60 μm; minimal scan time of 3 min) were provided to get reliable quantitative CT. The CTN (in Hounsfield unit [HU]) in each voxel inside each contoured ROI was specified as the mean CTN per tumor using the software tool. In order to ensure that the position of nude mice with no change, the CT scan bed was the same as that used in the PET scan.

Integrated PET/CT images were then reconstructed by the Inveon software (Siemens Medical Solutions) based on their scan layer thickness: ^18^F-FLT PET image of 0.78 mm slice thickness, 0 mm interval and CT images of 0.08 mm slice thickness, 0.23 mm interval. The thickness of two PET layers (1.56 mm) was similar to that of five CT layers (1.55 mm).

### Immunohistochemistry studies

After excision, tumors were fixed overnight in 10% formalin and embedded in paraffin blocks, from which 4 -um sections were cut for immunohistochemical staining. To measure tumor proliferation, slides were incubated with mouse monoclonal antibodies against Ki-67 MIB-1 (dilution 1:200; Dianova; Hamburg; Germany). For Ki-67 staining, 4–5 non-overlapping fields of non-necrotic areas were randomly selected in each ROI. The number of Ki-67 positively labeled cells was collected as the positively-stained nuclei of 200 cells in each field, derived from images of stained sections at 100× magnifications. The labeling index (LI) of Ki-67 was calculated as the total number of Ki-67 positively stained cells of all the 4–5 fields divided by 800 or 1000. An in-house software tool developed with Motic DSAssistant Lite (version R2013a; MathWorks, Natick, MA) was used to analyze the selected pathologic slices.

### Statistical Analysis

Statistical analysis was performed with SPSS software for Windows, version 17.0 (SPSS Inc.). The alterations in TV, uptake of ^18^F-FLT, CTN were assessed using the 2-tailed paired t test. All data were expressed as Mean ± standard deviation (SD). The differences with regard to tumor volume, the uptake of ^18^F-FLT and CTN among different groups were analyzed with one-way ANOVA followed by the Bonferroni post hoc test. Correlations between CTNs, ^18^F-FLT uptake changes and Ki-67 LIs were analyzed using linear regression analysis and the nonparametric Spearman rank test. A probability value of less than 0.05 was considered to denote statistical significance.

## Results

**Figure 1:**
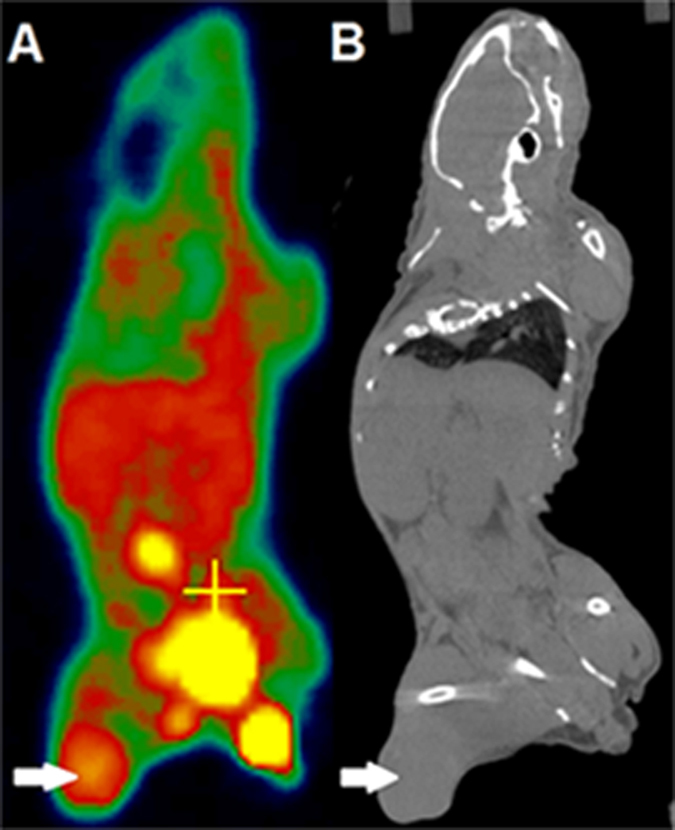
Whole body PET and CT imaging of the No. 2 mice in 3f/6d group. (**A**) ^18^F-FLT PET imaging (**B**) CT imaging. Arrows point to tumor.

All of the 120 mice (60 for each fractionation group) received the given irradiation, ^18^F-FLT micro-PET scans and micro-CT scans as planned. Figure 1 showed one example of the whole body PET and CT imaging of the No 2 mouse in 3f/6d group. The tumor lesion had a good uptake of ^18^F-FLT, while the normal tissue maintained a low level of uptake of the imaging probe. The high target-to-background ratios made the PET imaging contrasting and valuable (Fig. 1A). A faint outline of tumor could be observed in the CT imaging (Fig. 1B). Tumor volumes were evaluated every 3 days.

### Pathological Changes

**Figure 2:**
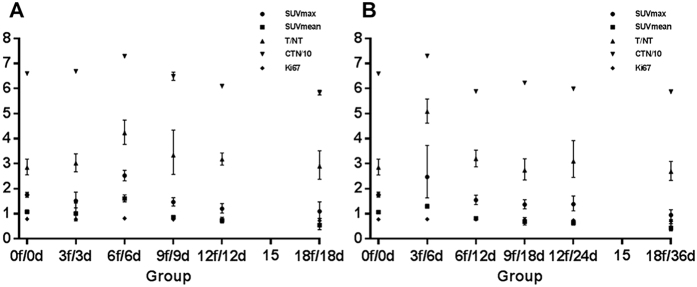
Dose-response curves for FLT uptake parameters, CTN changes, Ki67 LI in irradiated daily and every second group. In order to balance the four curves in the same figure, the ratio of CTN/10 was used to replace CTN (in HU) and the KI67 LI was expressed in decimal point format. (**A**) In irradiated daily group, both FLT uptake (SUVmax, SUVmean, T/NT ratio) and CTN increases were observed after 6 times of irradiation. (**B**) In irradiated every second day group, FLT uptake and CTN increased significantly after irradiation of 6Gy/3f compared with non-irradiated tumors.

Figure 2 showed the changes of Ki-67 LI among different treatment groups. The mean Ki-67 LI for non-irradiated tumors was 79.01% and 76.54%. A significant increase to 81.98% and 78.30% was observed after daily irradiation of 6 fractions in 6 days (12Gy/6f/6d) (P < 0.0001) and after every second day irradiation of 3 fractions in 6 days (6Gy/3f/6d) (P = 0.002), respectively. Taken together, these Ki-67 LI changes under different fractionation schemes validated the A549 xenograft model of accelerated tumor repopulation.

### Tumor Volume Change

Tumors experienced TV evaluation during the RT delivery every 3 days. No difference was found before irradiation within 12 sub-groups aimed to receive different dose (P > 0.05).

**Figure 3:**
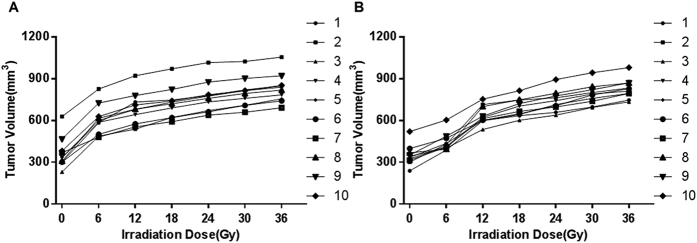
Dose–response curves for each tumor in 18f/18d group and 18f/36d group. (**A**) No significant shrinkage of TVs was noted during RT delivery for the 10 tumors in 18f/18d group. (**B**) For the 10 tumors in 18f/36d group, with accumulation of irradiation dose, no shrinkage of the TVs was observed.

Because only tumors in 18f/18d group and 18f/36d group received a radiation dose of up to 36Gy and whole course volume monitoring, we conducted a data analysis of TV changes for these two groups. Based on the TVs after irradiation, we draw dose–response curves for these two groups. When irradiation was completed, the mean (±SD) volumes of tumors were 828.76 ± 76.72 mm^3^ for 36Gy/18f/18d group, and 830.83 ± 102.77 mm^3^ for 36Gy/18f/36d group, respectively. The dose–response curves for every tumor’s growth were shown in Fig. 3. With accumulation of irradiation dose, no obvious shrinkage of the TV was observed.

When the irradiation was completed, TVs of different groups were also measured. We draw dose–response curves for different groups. Similar results were received in the end. With irradiation dose increasing, no shrinkage of the TV was noted.

### ^18^F-FLT PET parameters

Compared with non-irradiation tumors, the SUVmax, SUVmean and T/NT ratio increased significantly after 6 times of irradiation at irradiated daily group (2.527, Psuv_max_ < 0.001; 1.614, Psuv_mean_ < 0.001; 4.250, P_T/NT_ = 0.001) and 3 times of irradiation at irradiated ever second day group (2.494, Psuv_max_ = 0.001; 1.300, Psuv_mean_ = 0.027; 4.750 P_T/NT_ = 0.002, respectively), with treatment time prolonged, but decreased again at later time. The datas obtained from PET image of the two groups were shown in Tables 1 and 2, respectively.

### CTN change

For the non-irradiation tumors, no difference was found in CTN between irradiated daily and every second day group (P = 0.094). The CTN increased after several times of irradiation at both irradiated daily and every second day group with treatment time prolonged, followed by a persistent decrease at later time. Figure 2 showed the dose-response curves for CTN of two groups. CTN change for tumors of irradiated daily group was shown in Fig. 2A. Compared to the mean CTN of non-irradiated tumors (66.009 HU, 95% confidence interval [CI] 65.961–66.056 HU), significant increases were observed after 6 times of irradiation (72.968HU, 72.874–73.063HU, P < 0.0001). Figure 2B showed CTN increased after irradiation of 6Gy/3f at irradiated every second day group (73.034HU, 95%CI 72.695–73.373HU, P < 0.0001) compared with non-irradiated tumors.

### Correlation Analysis for FLT uptake, CTN change, TV and Ki-67 LI

Results of the linear regression analysis found that changes in the standard deviation of ^18^F-FLT uptake parameters (SUVmax, SUVmean) depended linearly on the changes of the average Ki-67 LI for daily irradiation group. Similar results were also found in the irradiated every second day group (Table 3). Strong correlations were also found between Ki-67 LI and CTN (Table 3). No marked relationships were observed between Ki-67 LI and post-irradiation TVs of different groups (P > 0.05).

In addition, strong correlations were found between CTN and SUVmax, SUVmean, T/NT ratio in two groups (Table 4). Comparison test failed to detect significant relationships between TV changes and CTNs as well as ^18^F-FLT uptake parameters. In sum, both ^18^F-FLT uptake parameters and CTN correlated strongly with immunohistochemical markers of proliferation, consistent with findings of accelerated tumor repopulation.

## Discussion

As we all know, the anatomic change, especially the volumetric change is the major interfractional variation observed during the course of RT delivery for solid tumors. CT image has long been used to detect the tumor shape and volume change during or after RT and can help monitor as well as guide RT planning for tumors. But what we should notice is that TV changes are sometimes too small or happen too late to timely provide accurate and useful information of tumor growth for RT re-planning. More and more studies have discovered the potential of CT to observe the molecular biological features of tumors^15^,^18^,^19^,^20^. The study conducted by Mayer *et al*. revealed that radiation treatments reduced the average CT number of lung cancer (P < 0.001) and this reduction seemed to be associated with tumor local control at 10 months^20^. In addition, Feng *et al*.^15^ found a fair correlation between CTN reductions and radiation doses for a subset of Head and Neck cancer (HNC) patients. Similar results had also been observed by Xu *et al*.^18^, that the CTN change during radiotherapy was dose dependent and was measurable for NPC, implying that the CTN might be an indicator to monitor tumor growth during treatment.

Previous studies have found a significant correlation between Ki-67 LI and ^18^F-FLT uptake before and during RT delivery in non-small cell lung cancer (NSCLC)^21^. Controversy remained over the value of CTN in monitoring tumor proliferation. Thus, a comparative study based on pathological information was conducted in this work to observe the relationship between tumor volume regressions, ^18^F-FLT uptake changes, CTN changes, and delivered radiation dose during the courses of RT. This research was designed to explore the diagnostic value of CTN as a promising imaging surrogate of tumor proliferative response to fractionated radiotherapy in NSCLC.

Data analysis of Ki-67 LI in different groups revealed that accelerated tumor cell repopulation happened when the dose reached 12Gy/6f/6d in irradiated daily group and 6Gy/3f/6d in irradiated every second day group. These change patterns of proliferation markers were in remarkably good agreement with the ^18^F-FLT uptake of the repetitive PET/CT scans. Similar trend has been observed in CT scan images with tumor repopulation being induced by the same irradiation schedules (6f/6d and 3f/6d). But we thought a more frequent CT scans or a longitudinal measurements might help find the very exact point of significant CTN increase in the follow-up studies. The ongoing studies conducted by Allen *et al*. at the department of Radiation Oncology of Medical College of Wisconsin tend to overcome this limitation with dual energy CT which has the capacity to amplify treatment response during RT delivery.

Result of our study also figured out that, tumor volume failed to shrink and continued to grow slowly during the RT delivery. The trend of TV changes did not correlate well with Ki-67 LI changes. Comparison test also failed to detect a good correlation between TV change and CTN as well as ^18^F-FLT uptake during the present study time frame. This result was in coincidence with the previous reports^11^,^15^,^18^. Mayer *et al*. have ever examined the radiation-induced changes in standard deviation of the CTN and failed to find a consistent relative changes in tumor volumes and CTN (R^2^ = 0.02)^20^. It has also been observed experimentally that clonogen number may or may not scale in proportion to changes in tumor volume^22^. Furthermore, Professor Johnson ever presumed that the intrinsic heterogeneity of radio sensitivity might be responsible for the nonlinear relationship to volume^22^. Besides, TV change could be detected by present simple diameter measurement only when cellular depletion exceeds a threshold. This indicated that TV monitoring was neither the best way to evaluate the treatment outcome in time nor the proper way to monitor the proliferation status of tumor cells during RT delivery.

One major limitation of the present study was the natural difference between animal model and patients, which obscured the universality and accuracy of this conclusion. In addition, as mentioned earlier, we only randomized the tumors of both the irradiated daily and irradiated every second day group into 6 subgroups (0f/3f/6f/9f/12f/18f) in this study. Lack of precise grouping gave us limited data to analyze the CTN change and this might affect the eventual results. Some other RT delivery proposals with different radiation parameters such as the linear energy transfer, the total dose, the dose per fraction and the time interval between two subsequent fractions might also lead to diverse results. Apart from geometrical restrictions, qualitative quantitative control of gray level (CT number; water corresponds to 0 Hounsfield units) windows as well as some instrumental uncertainties of CTN might have affected this study’s statistical analysis. Another limitation of our study was that the relatively small sample size included which precluded concluding that CTN or FLT uptake should replace simple volumetrics as indicators of tumor cell status. What also limited the generalization of CTN use was that no standard cutoff values had been figured out to indicate tumor biologically relevant changes. Moreover, the clinical standard for NSCLC is chemoradiotherapy, the addition of chemotherapy might influence the repopulation behavior of tumor. Thus the preclinical results may not be translatable to the clinical situation as other treatment regimens are applied. Consequently, it is important to confirm our findings in larger study cohorts through a way that would allow cross validation and pooled analysis of the results. Only one kind of NSCLC cell line-A549 was used in this study, so further investigation is warranted to validate the theory in other cell lines and animal models, as well as in clinical practice.

## Conclusion

This pilot study suggested that changes in CT number might provide an early indicator of lung tumor response to radiation and help monitor the tumor cell growth status in order to detect the accelerated tumor cell repopulation. However, more strict quality-controlled prospective studies with large sample size which could permit every specific individual’s data to examine tumor biology on a casebycase and treatment-by-treatment basis if possible, were in need to verify this conclusion in other tumor models and in clinics.

## Additional Information

**How to cite this article**: Zhang, X. *et al*. In-vivo Comparison of ^18^F-FLT uptake, CT Number, Tumor Volume in Evaluation of Repopulation during Radiotherapy for Lung cancer. *Sci. Rep.* **7**, 46000; doi: 10.1038/srep46000 (2017).

**Publisher's note:** Springer Nature remains neutral with regard to jurisdictional claims in published maps and institutional affiliations.

## Figures and Tables

**Table 1:** ^18^F-FLT uptake parameters of tumors in irradiated daily group.

Group	SUVmax			SUVmean			T/NT ratio		
	mean	SD	95%CI	mean	SD	95%CI	mean	SD	95%CI
0Gy/0f/0d	1.757	0.094	1.658–1.855	1.075	0.056	1.017–1.134	2.864	0.302	2.546–3.181
6Gy/3f/3d	1.542	0.306	1.221–1.864	1.063	0.389	0.654–1.471	3.033	0.362	2.653–3.413
12Gy/6f/6d	2.527	0.207	2.311–2.744	1.614	0.389	1.468–1.759	4.250	0.440	3.788–4.712
18Gy/9f/9d	1.470	0.101	1.309–1.631	0.865	0.035	0.809–0.921	3.375	0.576	2.458–4.291
24Gy/12f/12d	1.212	0.156	1.019–1.405	0.742	0.092	0.629–0.856	3.183	0.195	2.941–3.426
36Gy/18f/18d	1.139	0.3	0.861–1.417	0.580	0.179	0.415–0.745	2.951	0.652	2.348–3.554

SUV, standardized uptake value; SUVmax, the maximum SUV; SUVmean, the mean SUV; T/NT ratio, tumor-to-normal tissue ratio; SD, standard deviation; CI, confidence interval; f, fraction.

**Table 2:** ^18^F-FLT uptake parameters of tumors in irradiated every second day group.

group	SUVmax			SUVmean			T/NT ratio		
	mean	SD	95%CI	mean	SD	95%CI	mean	SD	95%CI
0Gy/0f/0d	1.760	0.094	1.661–1.859	1.069	0.051	1.016–1.122	2.863	0.304	2.544–3.182
6Gy/3f/6d	2.494	0.407	1.482–3.507	1.300	0.032	1.221–1.380	4.750	0.732	2.933–6.568
12Gy/6f/9d	1.557	0.185	1.364–1.751	0.821	0.047	0.771–0.870	3.208	0.310	2.882–3.533
18Gy/9f/12d	1.377	0.174	1.195–1.560	0.695	0.142	0.546–0.844	2.763	0.375	2.369–3.157
24Gy/12f/24d	1.406	0.304	1.087–1.725	0.640	0.096	0.540–0.741	3.162	0.670 1.314	2.459–3.865
36Gy/18f/36d	0.963	0.181	0.773–1.153	0.455	0.119	0.330–0.580	2.703	0.361	2.325–3.082

SUV, standardized uptake value; SUVmax, the maximum SUV; SUVmean, the mean SUV; T/NT ratio, tumor-to-normal tissue ratio; SD, standard deviation; CI, confidence interval; f, fraction.

**Table 3:** Relationship between Ki-67 LI and ^18^F-FLT uptake parameters as well as CTN.

Correlation	Ki67 LI			
	Irradiated daily group		Irradiated every second day group	
	r	P	r	P
SUVmax	0.838	<0.0001	0.747	<0.0001
SUVmean	0.855	<0.0001	0.771	<0.0001
T/NT ratio	0.321	0.073	0.287	0.105
CTN	0.891	<0.0001	0.886	<0.0001

LI, labeling index; SUV, standardized uptake value; SUVmax, the maximum SUV; SUVmean, the mean SUV; T/NT ratio, tumor-to-normal tissue ratio; CTN, CT number.

**Table 4:** Relationship between CTN and ^18^F-FLT uptake parameters.

Correlation	CTN			
	Irradiated daily group		Irradiated every second day group	
	r	P	r	P
SUVmax	0.799	<0.0001	0.853	<0.0001
SUVmean	0.849	<0.0001	0.919	<0.0001
T/NT ratio	0.375	0.029	0.334	0.058

CTN, CT number; SUV, standardized uptake value; SUVmax, the maximum SUV; SUVmean, the mean SUV; T/NT ratio, tumor-to-normal tissue rati.
